# The metabolic/pH sensor soluble adenylyl cyclase is a tumor suppressor protein

**DOI:** 10.18632/oncotarget.10056

**Published:** 2016-06-15

**Authors:** Lavoisier Ramos-Espiritu, Ana Diaz, Charlee Nardin, Anthony J. Saviola, Fiona Shaw, Tamar Plitt, Xia Yang, Jedd Wolchok, Edyta C. Pirog, Garrett Desman, Andrea Sboner, Tuo Zhang, Jenny Xiang, Taha Merghoub, Lonny R. Levin, Jochen Buck, Jonathan H. Zippin

**Affiliations:** ^1^ Department of Pharmacology, Weill Cornell Medical College, New York, NY, USA; ^2^ Department of Dermatology, Weill Cornell Medical College, New York, NY, USA; ^3^ Service de Dermatologie, Centre Hospitalier Universitaire, Besançon, France; ^4^ Ludwig Collaborative and Swim Across America Lab, Memorial Sloan Kettering Cancer Center, New York, NY, USA; ^5^ Department of Medicine and Ludwig Center, Memorial Sloan Kettering Cancer Center, New York, NY, USA; ^6^ Department of Pathology and Laboratory Medicine, Weill Cornell Medical College, New York, NY, USA; ^7^ Department of Pathology and Laboratory Medicine, Icahn School of Medicine at Mount Sinai, New York, NY, USA; ^8^ The Institute for Computational Biomedicine, Weill Cornell Medical College, New York, NY, USA; ^9^ Genomics Resources Core Facility, Weill Cornell Medical College, New York, NY, USA; ^10^ Meyer Cancer Center, Weill Cornell Medical College, New York, NY, USA

**Keywords:** sAC, cAMP, tumor suppressor, microdomain, metabolic sensor

## Abstract

cAMP signaling pathways can both stimulate and inhibit the development of cancer; however, the sources of cAMP important for tumorigenesis remain poorly understood. Soluble adenylyl cyclase (sAC) is a non-canonical, evolutionarily conserved, nutrient- and pH-sensing source of cAMP. sAC has been implicated in the metastatic potential of certain cancers, and it is differentially localized in human cancers as compared to benign tissues. We now show that sAC expression is reduced in many human cancers. Loss of sAC increases cellular transformation *in vitro* and malignant progression *in vivo*. These data identify the metabolic/pH sensor soluble adenylyl cyclase as a previously unappreciated tumor suppressor protein.

## INTRODUCTION

cAMP signaling pathways have numerous and sometimes conflicting roles in human cancers [1]. cAMP levels result from a balance between production by adenylyl cyclases and catabolism by phosphodiesterases (PDEs). Some cancers (e.g., cancers of endocrine tissues [2, 3]) are induced by the activation of adenylyl cyclases; however, in other cancers, PDEs are upregulated [4]; therefore, either activation or inhibition of cAMP levels can promote tumorigenesis depending on the tissue. cAMP can inhibit signaling pathways important for cancer. For example, cAMP can either activate or inhibit (MAPK) pathway proteins essential for tumorigenesis via either exchange protein activated by cAMP (EPAC) stimulation of Rap or protein kinase A (PKA)-dependent inhibition of RAF1 or BRAF, respectively [1, 5–9]. While numerous studies have identified genetic alterations or aberrant regulation of PDEs or proteins upstream of cAMP, there is little known about the differential expression of the sources of cAMP, the adenylyl cyclases, in cancer.

Mammalian cells possess two families of adenylyl cyclase, transmembrane adenylyl cyclases (tmACs, ADCY1-9) and soluble adenylyl cyclase (sAC, ADCY10). Unlike tmACs, which are permanently tethered to the plasma membrane, sAC is present throughout the cytoplasm and is localized within the mitochondria and nucleus [10, 11]. tmACs are responsive to heterotrimeric G proteins while sAC is a bicarbonate ion (HCO_3_), calcium ion and ATP sensor [12–14] and the regulation of sAC-like adenylyl cyclases by HCO_3_^−^/calcium/ATP is conserved from cyanobacteria to man [13, 15]. Because HCO_3_^−^ concentration reflects changes in intracellular pH, sAC is able to respond to changes in pH and previous reports have demonstrated that sAC can initiate cellular changes to return pH to normal physiological levels [16, 17]. Intracellular HCO_3_^−^ is also in equilibrium with CO_2_, and because metabolism leads to the production of ATP and CO_2_, regulation of sAC by ATP and HCO_3_^−^ allows this enzyme to function as a metabolic sensor [14, 18]. In multiple mammalian cell types, sAC activity reflects nutritional status linking metabolism with key biological processes including cellular motility [19], insulin secretion [14], and neuronal activity [20]. Furthermore, in a signaling cascade conserved from yeast to mammals, mitochondrial sAC senses metabolically produced CO_2_ via HCO_3_^−^ to regulate oxidative phosphorylation [21, 22]. These studies establish sAC as a pH/metabolic sensor in mammalian cells [14, 16, 17, 21–24]. As a pH/metabolic sensor, sAC activity is poised to reflect both extracellular and intracellular changes normally encountered by cells during transformation and, therefore, sAC has the potential to influence tumorigenesis.

The localization of sAC within a cell changes as the cell progresses through mitosis and transitions between states of growth and differentiation [11, 25]. This dynamic localization implies sAC has multiple roles during cellular growth and differentiation, and it suggests sAC activity may impact proliferative diseases such as cancer. In fact, sAC localization patterns in certain tumors differ from normal tissues [26–29]. Furthermore, studies have suggested sAC plays a role in the metastatic progression of certain human cancers [26, 30]. However, the role of sAC in primary tumorigenesis has not yet been studied.

We now demonstrate that sAC expression is reduced in numerous human cancers and that loss of sAC stimulates tumorigenesis in mammalian cells both *in vitro* and *in vivo*. Loss of sAC results in activation of the MAPK pathway, which does not activate tumorigenesis on its own. However, when sAC loss is complemented with known oncoproteins or mutagens, cellular transformation is accelerated and enhanced. These data define sAC as a tumor suppressor protein.

## RESULTS

### sAC expression is diminished in human cancers

**Figure 1:**
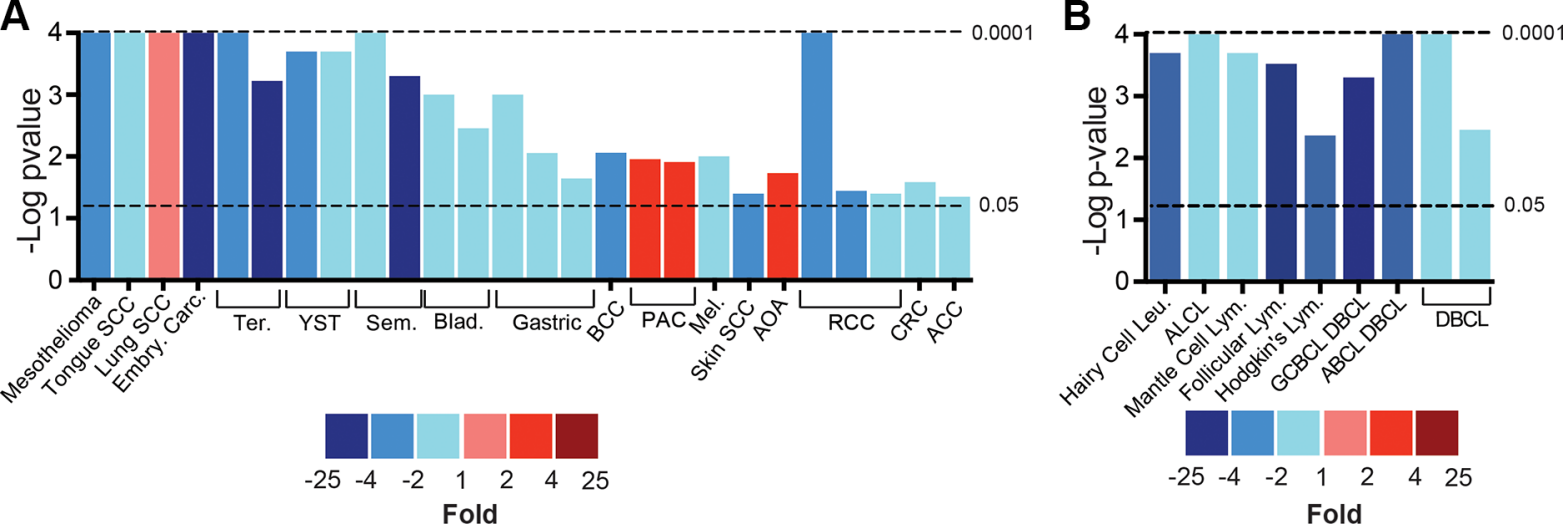
Expression of sAC mRNA is diminished in human cancers (**A**–**B**) sAC differential mRNA expression in solid (A) and hematologic (B) human cancers compared to normal controls. Bar height represents the negative Log 10 of the *P* value of the comparison and the color indicates the fold change of sAC mRNA level relative to normal controls (blue colors indicate diminished expression and red colors indicate elevated expression). Data collected from Oncomine as described in the methods. SCC = squamous cell carcinoma. Embry. = embryonic. Carc. = carcinoma. Ter. = teratoma. YST. = yolk sac tumor. Sem. = seminoma. Blad. = bladder. BCC = basal cell carcinoma. PAC = prostate adenocarcinoma. Mel. = melanoma. AOA = anaplastic oligoastrocytoma. RCC = renal cell carcinoma. CRC = colorectal carcinoma. ACC = adenoidcystic carcinoma. Leu. = leukemia. Lym. = lymphoma. ALCL = anaplastic large cell lymphoma. GCBCL = germinal center B-Cell lymphoa. DBCL = diffuse B-Cell lymphoma. ABCL = activated B-Cell like.

sAC localizes to distinct intracellular signaling microdomains [10, 11, 31] and is differentially expressed in tumor cells as compared to normal tissue [25, 27, 29, 32], but a comprehensive evaluation of sAC expression in human cancers has not yet been performed. Using publically available microarray databases, we compared sAC mRNA expression in human cancers and normal controls. Similar to the varied roles played by cAMP signaling in different cancers, sAC expression was significantly elevated in a few cancers but depressed in most. Consistent with previous studies identifying a positive role for sAC in prostate cancer [26], sAC expression was upregulated in prostate adenocarcinoma (PAC, Figure 1A). In contrast, sAC expression was significantly diminished in the majority of the solid tumor (Figure 1A and S1A and S1B) and hematologic (Figure 1B and S1C) malignancies analyzed.

**Figure 2:**
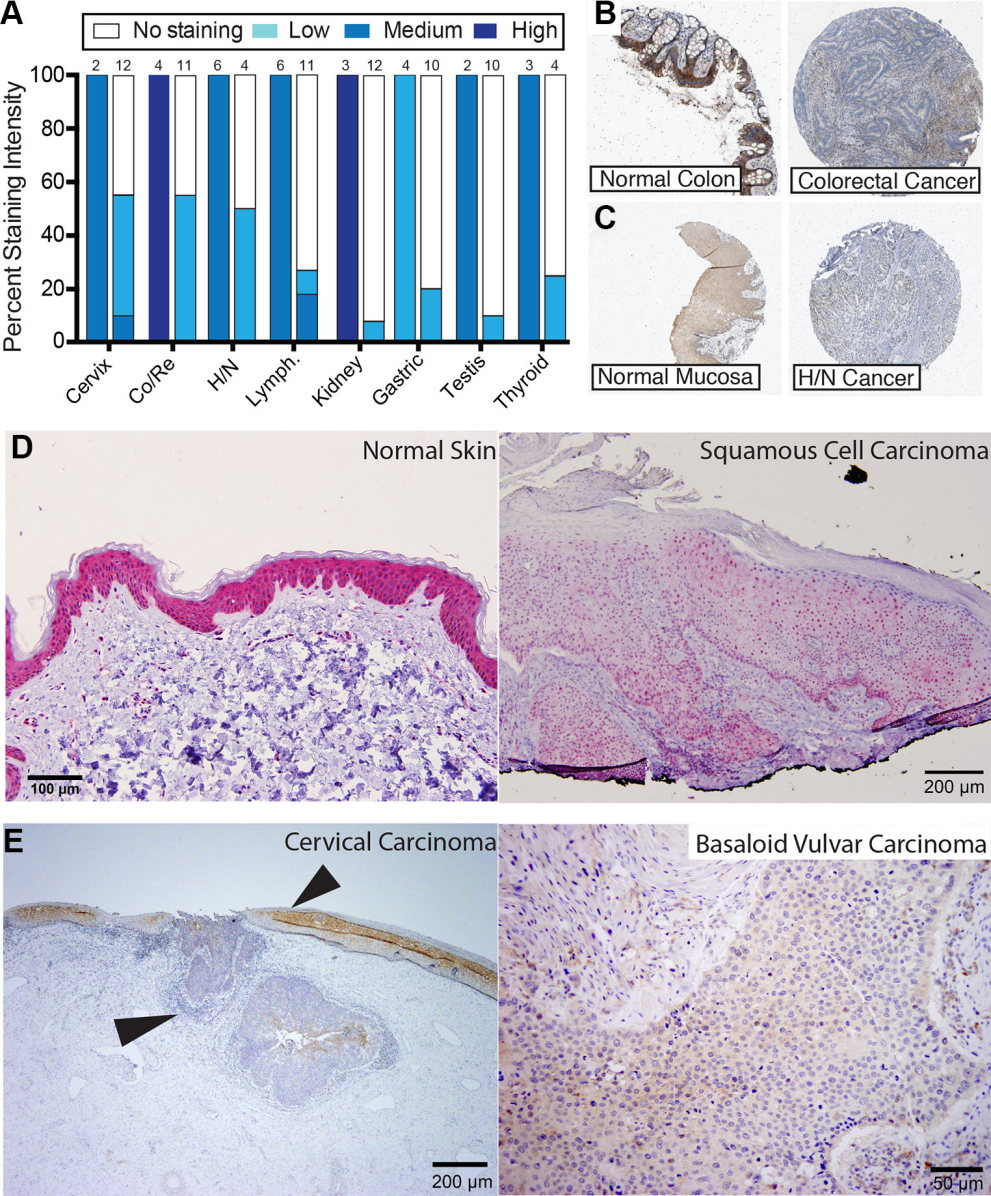
Expression of sAC protein is diminished in human cancers (**A**) Summary of immunohistochemical staining of normal human tissue (left bar) and human cancer (right bar) of data from the Human Protein Atlas using the anti human sAC antibody HPA017749. Color indicates staining intensity (white = no staining; light, medium, and dark blue = light, medium, and high staining intensity, respectively) and height of color is equivalent to the percentage of cases at that intensity. The number of cases analyzed in each group (*N*) is listed above the bar. (**B**) Representative example sAC expression (HPA017749, brown) in normal colon (left) and colorectal cancer (right) from Human Protein Atlas. In normal colon, sAC expression is mainly in the epithelial cells. (**C**) Representative example sAC expression (HPA017749, brown) in normal oral mucosa (left) and head and neck cancer (right) from Human Protein Atlas. In normal oral pharynx, sAC expression is mainly in the epithelial cells. (**D**) Normal human epidermis (left panel) and human squamous cell carcinoma of the genital skin (right panel) immunostained with anti-sAC antibody (R21; red chromagen). In normal skin, sAC expression is mainly in the epithelial cells. *N* = 10 (**E**) (Left panel) human cervical cancer (left arrow head) with adjoining normal cervix (right arrow head) and (right panel) basaloid vulvar cancer immunostained with anti-sAC antibody (R21; brown chromagen). Human tissue immunohistochemical staining was performed as previously described [25]. In normal cervix, sAC expression is mainly in the epithelial cells. *N* = 10 (D–E). In human skin staining there was 3+ staining in the epidermis (*N* = 6) and 1+ staining in squamous cell carcinoma (*N* = 6). In normal cervix there was 2+ staining and in cervical cancer (*N* = 10) there was 1 case with 0 staining of 100% of cells, 6 cases with 1+ staining of 100% of cells and 3 cases with 1+ staining of 90% of cells. In vulvar basaloid cancers (*N* = 6) all 6 cases showed 1+ staining of 100% of cells. SCC = squamous cell carcinoma. Ter. = Teratoma. YST = Yolk sac tumor. Sem. = Seminoma. Blad. = Bladder carcinoma. Gastric = Stomach adenocarcinoma. BCC = Basal cell carcinoma. PAC = Prostate adenocarcinoma. Mel. = Melanoma. AOA = Anaplastic oligoastrocytoma. RCC = Renal cell carcinoma. CRC = Colorectal carcinoma. ACC = Adenoid cystic carcinoma. Leu. = Leukemia. Lym. = Lymphoma. ALCL = Anaplastic large cell lymphoma. DBCL = Diffuse B cell lymphoma. ABCL = Activated B-Cell-Like. GCBCL = Germinal center B-Cell-Like. Co/Re = Colorectal. H/N = Head and Neck. Lymph. = Lymphoma.

To assess whether the reduction of sAC mRNA level was leading to changes in sAC protein expression, we examined data from the Human Protein Atlas [33]. Immunohistochemical staining from the Human Protein Atlas confirmed that in most human cancers where sAC expression was diminished, sAC protein level is decreased relative to normal tissue controls (Figure 2A). For example, immunohistochemical staining of sAC in colorectal and head/neck carcinoma cancers reveal reduced sAC protein in these cancers relative to normal tissue controls (Figure 2B and 2C). Downregulation of sAC protein in human cancers was independently confirmed using the anti-human sAC monoclonal antibody R21, which we generated in our laboratory and previously characterized in cells and in human skin [25]. R21 immunostaining demonstrated a decrease in sAC protein in squamous cell carcinomas (SCC) relative to adjacent normal tissues (Figure 2D and 2E). Examination of human cancer tissue sections at low magnification demonstrated the abrupt transition of sAC expression from normal squamous epithelium to carcinoma (Figure 2E, left panel, arrows).

### Loss of sAC activity facilitates cellular transformation *in vitro*

**Figure 3:**
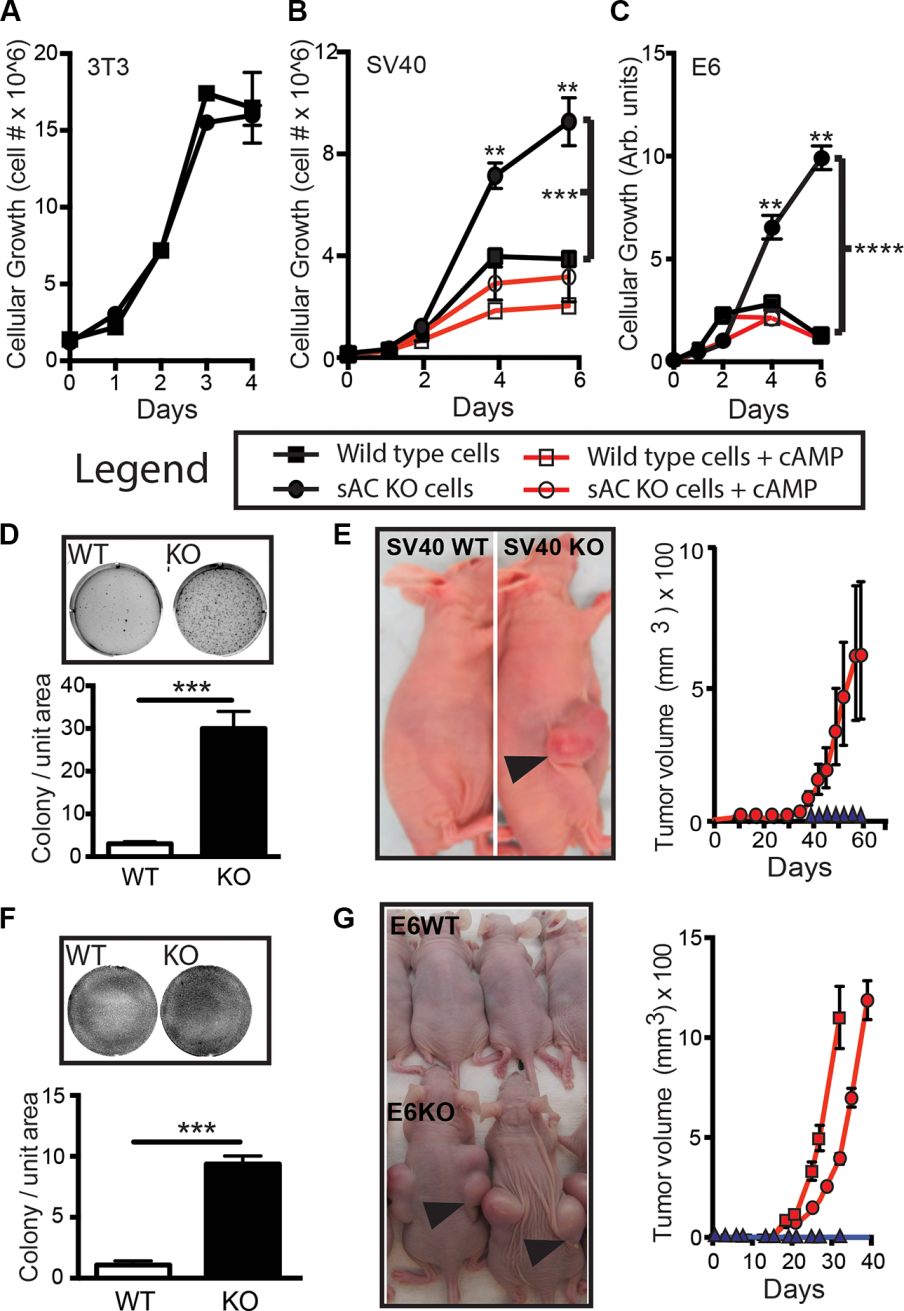
Loss of sAC activity enhances transformation *in vitro* (**A**–**C**) Cell growth of WT (squares) and sAC KO (circles) (A) 3T3, (B) SV40 and (C) HPV16-E6 MEFs in the absence (closed symbols, black lines) or presence (open symbols, red lines) of membrane permeable cAMP (B, 100 μM and C, 500 μM) for the number of days indicated. A-C, data presented is *N* = 4 for each data point. Experiments performed at least three times on two sets of independently produced cell lines. Legend below (A–C) identifies conditions. Notice: 3T3 wild type and sAC KO cells are contact inhibited. Repeated measures ANOVA. **P < 0.01, ***P < 0.001, ****P < 0.0001. *P* Values indicated by brackets represent treatment group comparisons. (**D**) Colony formation (field image, upper panel) and quantitation (lower panel) of colony number of SV40 WT (WT, white bar) and sAC KO (KO, black bar) MEFs. *N* = 3 for each data point. Experiments performed at least three times on two sets of independently produced cell lines (**E**) Left panel, image of tumor formation in nude mice from WT (SV40 WT) and sAC KO (SV40 KO) after 60 days (arrow indicate tumors). Right panel, recorded tumor volume over time after injection with SV40 KO (5 million cells, red circles). SV40 WT (5 million cells, blue triangles) MEFs did not develop tumors. *N* = 10 for each genotype (**F**) Colony formation (field image, upper panel) and quantitation (lower panel) of colony number of E6 WT (WT, white bar) and sAC KO (KO, black bar) MEFs. *N* = 3 for each data point. Experiments performed at least three times on two sets of independently produced cell lines (**G**) Left panel, image of tumor formation in nude mice from WT (E6WT) and sAC KO (E6KO) after 30 days (arrows indicate tumors). Right panel, recorded tumor volume over time after injection with E6KO (red lines, 5 million cells [squares], 2 million cells [circles]). E6WT (black lines, 5 million cells, [triangles]) MEFs did not develop tumors. *N* = 10 for each genotype. Of note, while WT cells rarely formed colonies in soft agar, when WT colonies did form they were smaller on average than sAC KO colonies. Tumor xenografts were measured weekly using calipers, and a veterinary pathologist visually confirmed tumors after euthanasia. Error is represented as SEM. (D, F) Student's *t*-test, ***P < 0.001. F or E and G, experiment was reproduced using a distinct set of cell lines with identical results (data not shown).

The human cancer data suggested that loss of sAC may be important for carcinogenesis. Therefore, we asked if loss of sAC was sufficient to induce transformation in an *in vitro* context. We used the 3T3 method to generate immortalized embryonic fibroblasts (MEFs) from C57Bl/6 backcrossed sAC KO mice and WT littermates (Figure S2A). As expected, sAC KO MEFs generated less cAMP than WT MEFs, and the cAMP produced in sAC KO MEFs was not sensitive to sAC specific inhibitors (Figure S2B and S2C). Similar to WT MEFs, even after 90 passages in culture, sAC KO 3T3 MEFs did not transform; i.e., they still grow in a contact inhibited manner (Figure 3A) and can not grow in semi-solid agar. Because cellular transformation often requires complementing oncogenic changes [34], we tested whether addition of oncogenes could transform sAC KO primary fibroblasts. Introduction of the SV40 Large T antigen viral oncogene into primary WT fibroblasts led to their immortalization, but not transformation, even after 30 passages. In contrast, introduction of SV40 Large T was sufficient to transform sAC KO primary fibroblasts within a few passages (Figure 3) [35]. sAC KO SV40 MEFs, but not WT SV40 MEFs, exhibited a loss of contact inhibition (Figure 3B and 3D, upper panel), grew in semi-solid agar (Figure 3D, lower panel) and formed tumors in nude mice (Figure 3E). These results were reproduced using MEFs generated via a separate mating (data not shown). We further confirmed these results using a second independent oncogene. Because sAC expression was significantly diminished in human cancers driven by oncogenic human papilloma virus (HPV) (e.g., head and neck cancer, cervical cancer, and squamous cell carcinoma of the skin (Figures 1 and 2)), we introduced HPV16 E6 oncogene into both WT and sAC KO primary fibroblasts. Similar to our observations following introduction of SV40 Large T, HPV16 E6 induced loss of contact inhibition (Figure 3C and 3F, upper panel), growth in semi-solid agar (Figure 3F, lower panel) and formation of tumors in nude mice (Figure 3G) in sAC KO E6 MEFs, but not in WT E6 MEFs. The transformed phenotypes of sAC KO SV40 and E6 MEFs were reverted by treatment with membrane permeable cAMP (Figure 3B and 3C) consistent with the transformed phenotypes being dependent upon the absence of sAC. Thus, sAC loss complements established oncogenes to increase cellular transformation *in vitro*.

### Loss of sAC leads to activation of the MAP Kinase signaling pathway

**Figure 4:**
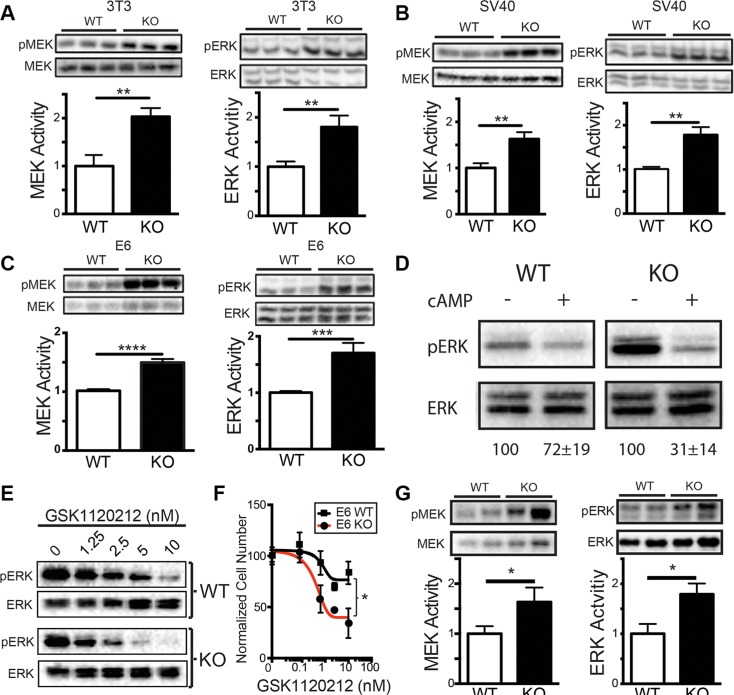
Loss of sAC activity leads to MAPK pathway activation (**A**–**C**) MAPK activity in mouse cells following serum starvation. Western blot and quantitation (normalized to WT) of pMEK/MEK (MEK Activity, 43 kDa, left panels) and pERK/ERK (ERK Activity, 44/42 kDa, right panels) in WT (white bars) and sAC KO (black bars) (A) 3T3 MEFs, (B) SV40 MEFs, and (C) E6 MEFs. For A-C, *N* = 3. (**D**) MAPK activity in 3T3 MEFs following the addition of Sp-8-CPT-cAMPs (+, 500 μM) or DMSO control (−) for 15 minutes. Data is a Western blot of pERK/ERK (44/42 kDa). Representative figure of an experiment performed in triplicate. Below Western blot is the average band quantitation (across all experiments) relative to DMSO control (set to 100%). (**E**) Western analysis of pERK (44/42 kDa) and total ERK (44/42 kDa) in WT (WT, top) and sAC KO (KO, bottom) MEFs following incubation for 1 hour with the MEK inhibitor GSK1120212 at the concentration indicated. Representative figure of an experiment performed three times. (**F**) Normalized cell number of WT (black lines, squares) and sAC KO (red lines, circles) MEFs following 72 hours of treatment with GSK1120212 at the concentrations indicated (*N* = 4). (**G**) Western blot and quantitation (normalized to WT) of pMEK/MEK (MEK Activity, 43 kDa, left panels) and pERK/ERK (ERK Activity, 44/42 kDa, right panels) in WT (white bars) and sAC KO (black bars) mouse epidermis. *N* = 3 (A–C, G) Student's *t*-test. error ± SEM. (F) Repeated measures ANOVA with post-hoc Sidak. Bracket indicates comparison of treatment groups. *P < 0.05, **P < 0.01, ***P < 0.001, ****P < 0.0001.

To better understand why sAC KO cells are more susceptible to transformation, we explored whether specific signaling pathways known to be important for transformation were elevated in sAC KO cells. The MAPK pathway, one of the most commonly activated signaling pathways in cancer, is regulated by cAMP signaling, and can complement both SV40 Large T and HPV E6 to induce transformation [34, 36]. Therefore, we explored whether the MAPK pathway was activated in sAC KO cells. Immortalized (3T3) and transformed (SV40, E6) sAC KO MEFs had elevated levels of both phosphorylated MEK and ERK compared to the corresponding WT MEF lines (Figure 4A–4C). Elevated MAPK activity in sAC KO cells was more sensitive to treatment with membrane permeable cAMP as compared to WT cells (Figure 4D) consistent with MAPK activation in sAC KO cells being due to the loss of cAMP. Activation of the MAPK pathway was not dependent on the presence of serum as sAC KO MEFs exhibit elevated MAPK signaling in the absence (Figure 4A–4C) or presence of serum (Figure S3). RNAseq and whole exome sequencing of both WT and sAC KO MEFs did not reveal any differential gene expression, gain-of-function mutation or deleterious mutation in any MAPK signaling or regulatory protein; therefore, loss of sAC expression must induce a post-transcriptional signaling change(s) leading to the activation of the MAPK signaling cascade. To test whether this elevated MAPK pathway activity was essential for sAC KO cells, we took advantage of a pharmacological inhibitor of MEK, GSK1120212. sAC KO MEFs were far more sensitive to the MEK inhibitor GSK1120212; ERK phosphorylation and cell growth was blocked to a greater extent and at lower doses in sAC KO MEFs relative to WT MEFs (Figure 4E and 4F).

Elevated MAPK signaling is a general phenomenon of sAC KO cells and tissues. Epidermal tissue derived from sAC KO adult mice had elevated levels of phosphorylated MEK and ERK as compared to tissue derived from WT mice (Figure 4G). Elevated MAPK activity in sAC KO tissues prompted us to explore whether sAC KO mice are more prone to tumorigenesis.

### sAC knockout mice exhibit enhanced tumorigenesis

Similar to sAC KO MEFs, which did not exhibit features of transformation without introduction of a complementing oncoprotein, we failed to observe spontaneous tumor formation in sAC KO mice. To supply a second “hit” to sAC KO mice, we employed a chemical carcinogenesis mouse model consisting of a combination of 7,12-dimethyl-benz[a]anthracene (DMBA) and phorbol ester 12-O-tetradecanoylphorbol-13 acetate (TPA)(37). This is a classical model used to determine whether presumptive tumor suppressor proteins have a role in carcinogenesis *in vivo*. A single topical application of DMBA (initiation stage) [37, 38] is followed by repeated topical application of a tumor mitogen (TPA) (promotion stage) leading to benign papilloma growth [38]. The third stage, progression, is the malignant conversion of benign papillomas to invasive squamous cell carcinomas (SCC) [38]. This model is dependent upon a functioning MAPK signaling pathway [39], and when the MAPK cascade is hyperactivated, tumor formation is enhanced [40, 41]. Because sAC KO epidermal cells exhibit elevated MAPK signaling at baseline, we asked whether loss of sAC enhances tumorigenesis in the DMBA/TPA chemical carcinogenesis model.

**Figure 5:**
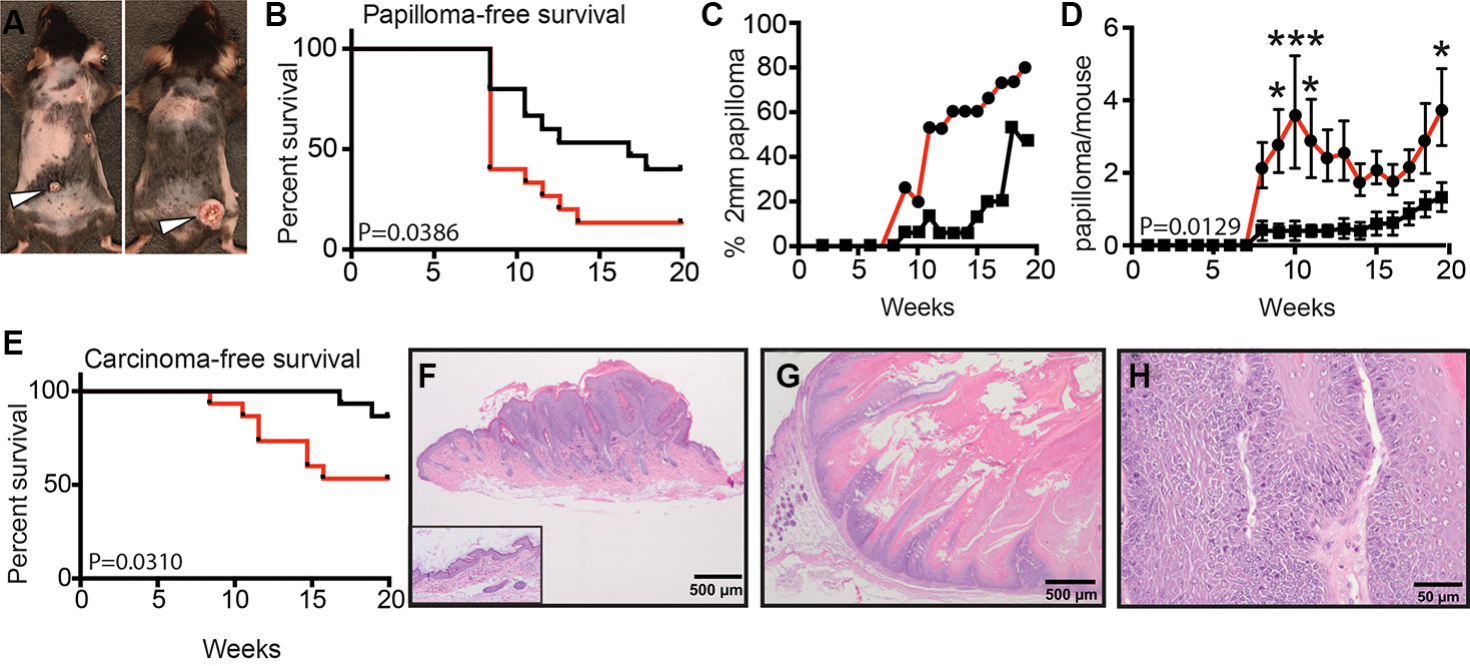
Loss of sAC enhances tumorigenesis in mice (**A**) Image of tumor formation in wild type (left) and sAC KO (right) mice. *N* = 15 mice for both wild type and sAC KO cohorts. (**B**) Papilloma free survival in wild type (black line) and sAC KO (red line) mice. *P* = 0.0386. (**C**) Percentage of wild type (squares, black line) or sAC KO (circles, red line) mice with sustained papilloma growth greater than 2mm per lesion. (**D**) Papilloma formation per mouse in wild type (squares, black line) and sAC KO (circles, red line) mice. *P* = 0.0129. error ± SEM (**E**) Carcinoma-free survival in wild type (black line) and sAC KO (red line) mice. *P* = 0.0310. (**F**–**H**) Hematoxylin and eosin stained sections of representative examples of mouse skin lesions. (F) Example of normal mouse epidermis (inset image) in comparison to an example of focal thickening of the epidermis (papilloma, main image) due to inappropriate keratinocyte growth following DMBA/TPA treatment. (G–H) Squamous cell carcinomas from a sAC KO mouse at low power (G) and at a higher magnification to demonstrate invasion (H). (B, E) *P* values indicates the significant difference between the treatment groups following Kaplan-Meier survival analysis. (D) Repeated measures ANOVA. *'s refer to statistical difference at a given time point as determined by Sidak post-hoc analysis of the repeated measures ANOVA. *P < 0.05, and ***P < 0.001.

While WT and sAC KO animals started developing stable papillomas at the same week (week 8) following DMBA treatment (Figure 5A and 5B), a higher frequency of sAC KO animals developed stable papillomas (93.3%) as compared to WT mice (60%) (Figure 5B and Table 1). This suggests that initiation of papilloma formation did not happen faster in sAC KO mice but promotion of papilloma growth did occur more frequently. In addition, sAC KO mice developed larger papillomas suggesting that the absence of sAC activity supports cell autonomous growth (Figure 5C and Table 1). Furthermore, sAC KO mice developed more numerous papillomas per mouse, again suggesting that loss of sAC activity drives tumor promotion (Figure 5D and Table 1). Finally, sAC KO mice developed SCCs earlier (week 8 for sAC KO vs. week 17 for WT mice) and in greater number (2 SCC in 2 WT mice [13.3%] vs. 11 SCC in 8 sAC KO mice [53.3%]) as compared to WT mice (Figure 5E, Figure S4, Table 1, and Table S1). As stated above, sAC KO mice do not develop spontaneous skin tumors and histologic evaluation of normal mouse epidermis does not reveal any overt structural difference between WT and sAC KO mice (Figure 5F, inset image). Histologic examination of papillomas (Figure 5F) and SCCs did not identify any overt cellular differences (e.g., number of mitoses, cellular architecture) between WT and sAC KO mice (Figure 5H and Figure S4); however, sAC KO SCCs were more invasive extending well below the dermal epidermal junction (Figure 5H).

**Table 1:** Comparison of tumor formation in wild type and sAC KO mice

	Wild type	sAC KO
**# Mice (N)**	15	15
**Average mouse weight (in g)**	25.3 ± 0.9	24.1 ± 0.4
**Total papillomas**	20	55 (*p* < 0.05)
**% mice with papillomas**	60%	93.3% (*p*< 0.05)
**Papillomas/mouse**	1.3 ± 0.4	3.7 ± 1.1 (*p* < 0.05)
**Average papilloma volume (mm^3^)**	10.3 ± 5.5	53.2 ± 19.7 (*p* < 0.05)
**Total papilloma volume in cohort (mm^3^)**	155	798
**Total carcinomas**	2	11

## DISCUSSION

Our studies demonstrate that loss of sAC facilitates transformation of cells *in vitro* and enhances the formation of tumors in mice. These results are consistent with studies examining the role of established tumor suppressor proteins in carcinogenesis [42]. Therefore, these data define sAC as a tumor suppressor protein. This is the first study to demonstrate that loss of an adenylyl cyclase can promote tumorigenesis.

cAMP signaling cascades have been shown to have both positive and negative effects on cancer cells [4, 43]. This seeming contradiction could be explained by the appreciation that physiological cAMP signaling is compartmentalized into spatially restricted microdomains [44] which allow this single second messenger to simultaneously regulate multiple, independent functions. In mammalian cells, cAMP microdomains are defined by two families of adenylyl cyclase; the hormone, and G protein-regulated transmembrane adenylyl cyclases (tmACs) and the bicarbonate, ATP, and calcium-regulated sAC. sAC represents the source of cAMP for microdomains in the cytoplasm, mitochondria, and nucleus [45]. Activating mutations in G proteins, which only stimulate tmACs [12], can lead to a subset of cancers [2, 3], and in particular cancers, namely prostate and breast adenocarcinoma, sAC activity seems to be required for metastasis or tumor invasion (Figure 1) [26, 30]. These data define instances where cAMP enhances carcinogenesis. In contrast, the observations that PDEs [4], which diminish cellular cAMP levels, are upregulated in numerous cancers, together with our findings that sAC is a tumor suppressor, define examples where low levels of cAMP are advantageous for carcinogenesis. These differences illustrate why it is no longer sufficient to simply study whole cell cAMP changes in cancer cells; to understand cAMP's role in carcinogenesis, it is imperative to study individual cAMP signaling microdomains.

It is intriguing to speculate why the expression of a metabolic and pH sensor, sAC, might be reduced in cancer. Cancers thrive in microenvironments with widely changing levels of nutrient availability and CO_2_/HCO_3_^−^/pH; therefore, diminished sAC activity, which would uncouple the control sAC has on MAPK activation, may provide a selective advantage for tumor cells. Future studies will be important to establish whether sAC expression levels correlate with different tumors at different sites of metastasis or different areas of tumor bulk (e.g., surface vs. core) exposed to unique metabolic and pH stress. In addition to reduced sAC expression (Figures 1 and 2), sAC has been shown to localize to distinct signaling microdomains in tumor cells as compared to normal tissue [25, 27]. Differential sAC immunohistochemical staining patterns are already proving useful as a cancer diagnostic adjunct [27, 29, 32], and our demonstration that sAC can function as a tumor suppressor suggests that these alterations in sAC-defined subcellular cAMP microdomains are important in cancer.

## MATERIALS AND METHODS

### Cell lines

Using C57Bl/6 wild type and sAC KO mice, we generated primary embryonic fibroblasts as previously described(1) and grown in DMEM + 10% FBS. Immortalization of fibroblasts (passage 3–4) was performed by either the 3T3 method (splitting cells 1:3 every three days) or transfection of a plasmid expressing the SV40 Large T Antigen or the HPV16-E6 viral oncogenes (gifts from the laboratories of Craig Thompson, Memorial Sloan Kettering laboratory, and Elliot Androphy, Indiana University School of Medicine, respectively) and splitting of cells when 80% confluent (1:3). Two sets of 3T3 and SV40 immortalized cell lines were produced from distinct embryos while two sets of HPV16-E6 immortalized lines were derived from a single transfection. All experiments in this manuscript were reproduced with duplicate sets of independently created cell lines.

### Western blot analysis

Equal numbers of cells were grown in 6 well to 70% confluency and either serum starved (DMEM + 0.5% BSA) or grown in normal serum. Cells or tissue (mouse epidermis) were lysed in NP40 lysis buffer or buffer provided by the company in the presence of phosphatase and protease inhibitors on ice and samples were normalized by protein level. Sensitivity of cell lines to MAPK inhibitors (GSK1120212 Selleck Chem) was assessed by incubating logarithmic growing cells in drug for the time indicated followed by either live cell counting (CyQuant, Life Tech) or for 1 hour followed by Western blot analysis. Antibodies used were: Phospho-ERK (Cell Signaling Technology #9101); ERK (Cell Signaling Technology #9102); Phospho-MEK (Cell Signaling Technology #9154); MEK (Cell Signaling Technology # 9122). Visualization and band quantification were done using a BioRad gel imager.

### *In vitro* transformation assays

Following plating of cells at 20–30% confluency, cell growth rate was assessed either by direct cell counting or by using the Cyquant fluorescent DNA binding assay (Life technologies) in 96 well format. Cells were assessed for growth in semi-solid agar by the Corning Clonal growth in Semisolid media protocol. The ability of cells to form tumors in nude mice were assessed by injecting either 2 or 5 million cells into both flanks of a nude mouse (Charles river) and mice were followed for xenograft growth 2–3 times a week. Mice were weighed and tumor volume was measured using calipers. Mice were sacrificed in accordance with approved IACUC protocols.

### Bioinformatic analysis

The Oncomine^™^ platform (Compendia Bioscience, Ann Arbor, MI) was used for dataset analysis and visualization. We examined all 202 available cancer versus normal datasets and 83 primary tumor versus metastatic tumor datasets using a cutoff of at least 1.5 fold difference and a *P* value ≤ 0.05 resulting in 26 datasets for tumor vs. normal and 5 datasets for primary vs. metastatic tumor. Datasets meeting these criteria were then examined using Prism (Graphpad Software, CA) for both proper control tissue and significance by Student's *t*-test. Representative individual datasets were visualized by scatterplots. Each dataset was also represented in a bar plot by showing the negative Log10 of *P* value between normal tissue and cancer. The bars of each dataset were color-coded according to fold change, with blue and red hues showing down- and up-regulation, respectively.

### *In vivo* chemical carcinogenesis

Animal studies were performed in accordance with approved IACUC protocols at WCMC. Genetic background is known to influence the rate of carcinogenesis, we backcrossed over 10 generations the sAC KO allele into the C57Bl/6 genetic background, as described in previous publications [14], which is known to be resistant to transformation [46]. This sAC KO mouse model has been confirmed to exhibit loss of sAC activity in numerous tissue types [14, 47, 48]. Two-stage carcinogenesis protocol was modified from the Cold Spring Harbor protocol 4837 (http://cshprotocols.cshlp.org). One week following removal of hair by hair clipper (Wahl CNT2-M) and Nair hair removal lotion, 100 μL of 7,12-Dimethylbenz[a]anthacene (DMBA, 400 nmol in acetone, Sigma D3254) was applied once to the back of 15 wild type (8 male and 7 female) and 15 sAC KO (7 male and 8 female) 6–7 week old mice. Starting one week later, 100 μL of 12-O-tetradecanoylphorbol 13-acetate, phorbol 12 myristate 13-acetate (TPA, 40 nmol in acetone, Sigma P8139) was applied once three times a week to the DMBA treated area. Mice were evaluated for tumor growth once weekly by a blinded dermatologist. Tumors/papillomas were recorded and mapped only if they were present for at least 2 weeks. At the completion of the experiment mice were euthanized, tumors were collected, and gross necroscopy was performed. Blinded histologic evaluation of epidermal tumors was performed by both an animal pathologist and a board certified dermatopathologist.

## Supplementary Materials Figures and Tables


