# Supplementary material for: The metabolic/pH sensor soluble adenylyl cyclase is a tumor suppressor protein
Source: Oncotarget. 2016 Jun 15;7(29):45597–607. doi: 10.18632/oncotarget.10056 (PMC5216745; doi:10.18632/oncotarget.10056)
Supplement: Supplementary file 1 [file oncotarget-07-45597-s001.pdf]

The metabolic/pH sensor soluble adenylyl cyclase is a tumor suppressor protein

Supplementary Materials

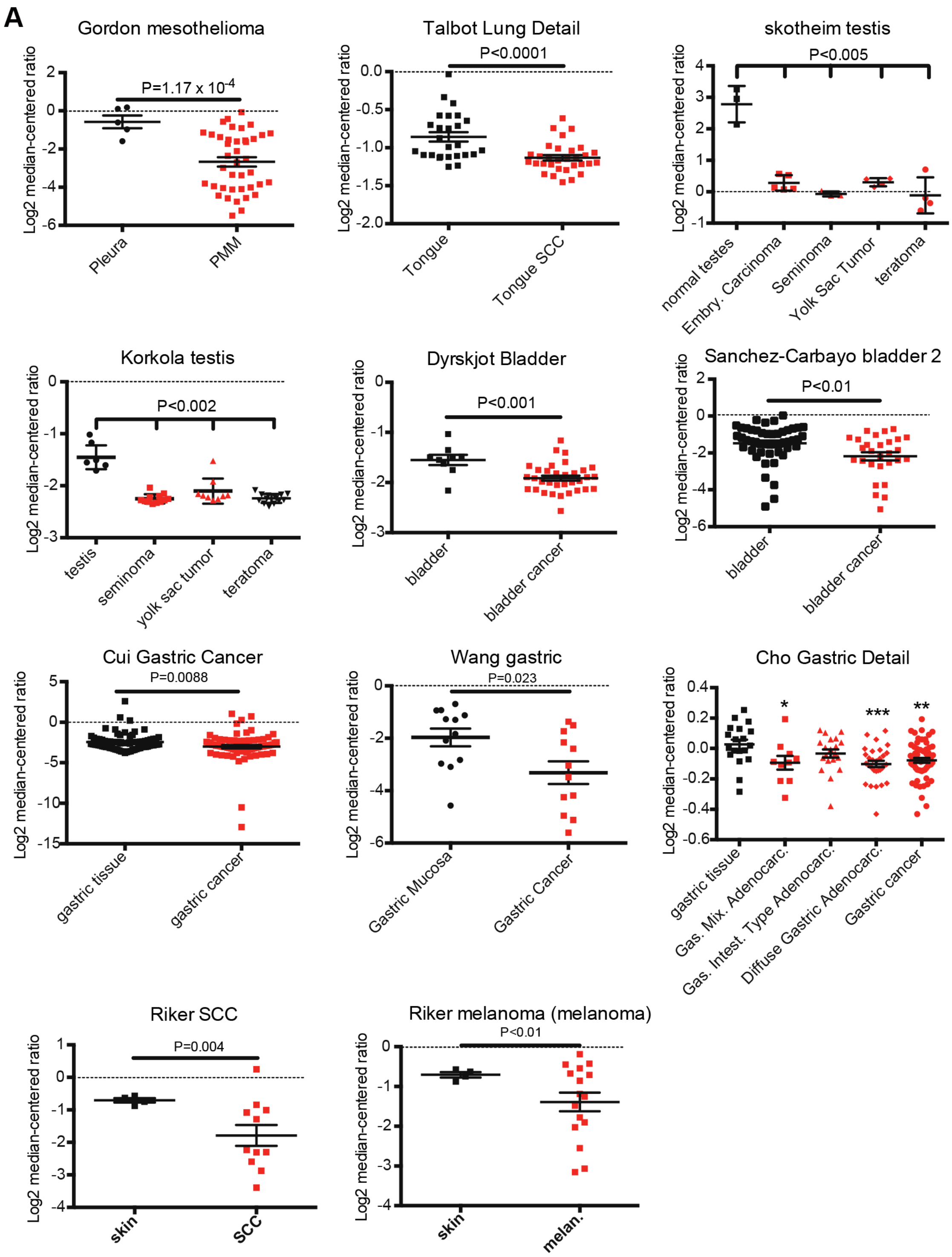

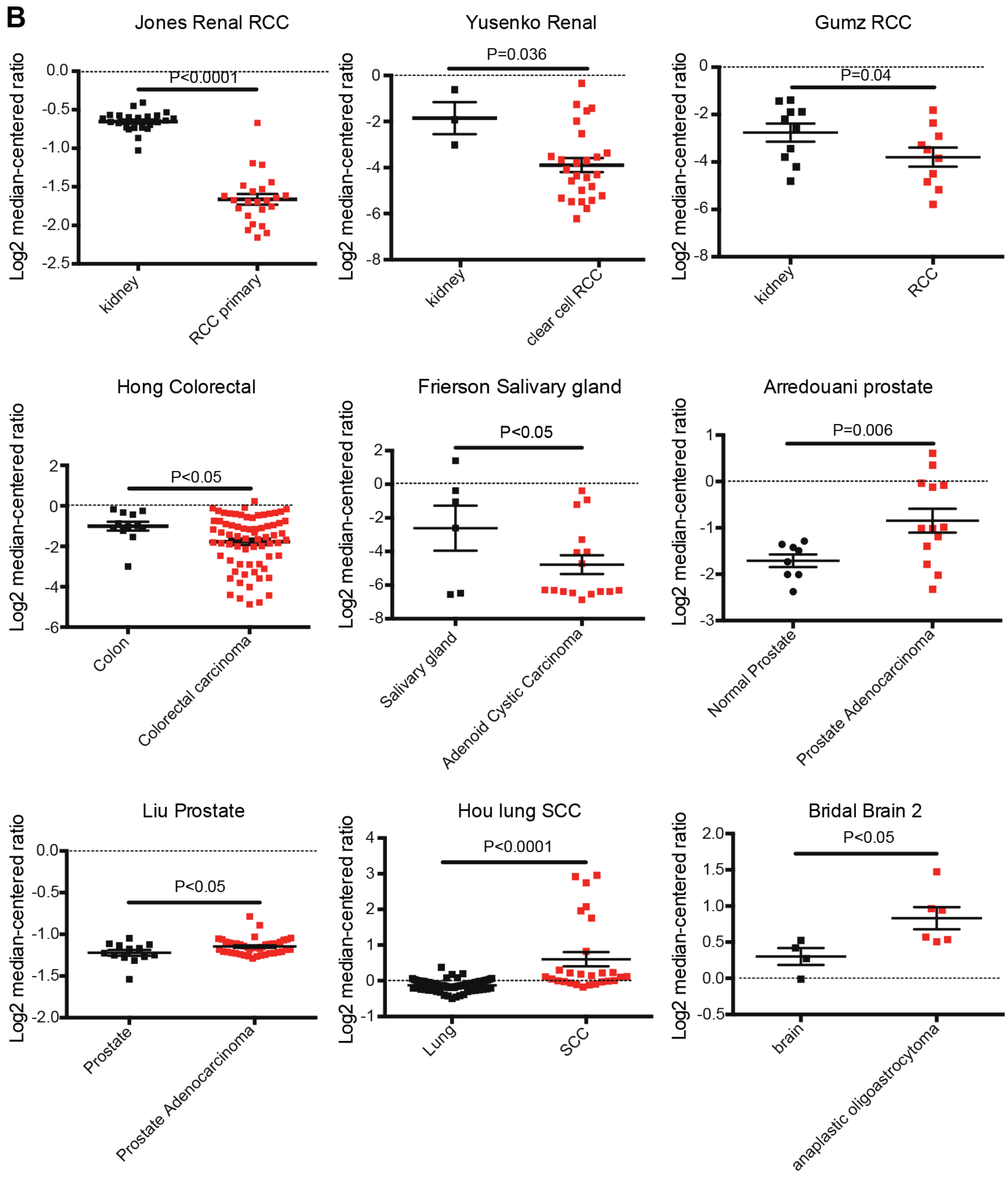

B
Jones Renal RCC
P<0.0001
Log2 median-centered ratio
0.0
-0.5
-1.0
-1.5
-2.0
-2.5
kidney
RCC primary
Yusenko Renal
P=0.036
0
-2
-4
-6
-8
kidney
clear cell RCC
Gumz RCC
P=0.04
RCC
Hong Colorectal
P<0.05
2
Colon
Colorectal carcinoma
Frierson Salivary gland
Salivary gland
Adenoid Cystic Carcinoma
Arredouani prostate
P=0.006
1
-1
-3
Normal Prostate
Prostate Adenocarcinoma
Liu Prostate
Prostate
Hou lung SCC
4
3
Lung
SCC
Bridal Brain 2
2.0
1.5
0.5
brain
anaplastic oligoastrocytoma

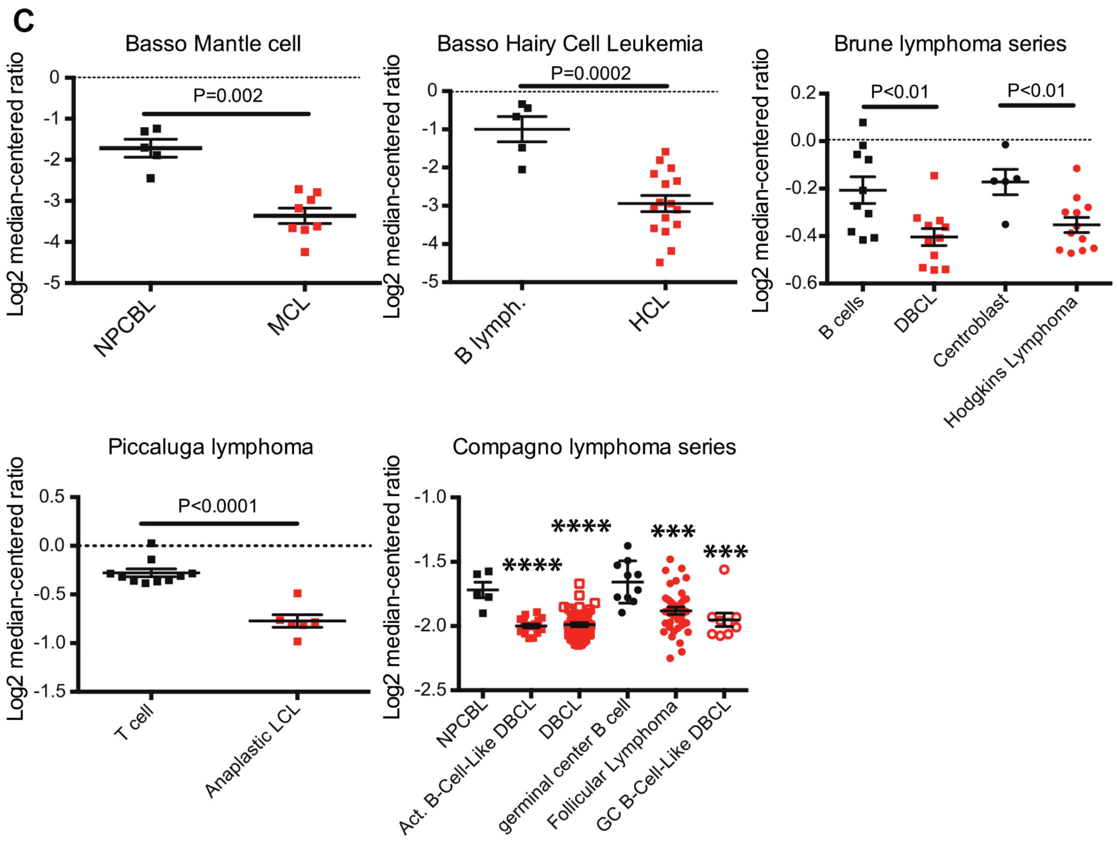

Supplementary Figure S1: sAC mRNA expression is decreased in human cancers as compared to normal controls. (**A**) Scatter plots of ADCY10 mRNA expression (represented as Log2 median centered ratio) in normal controls (black symbols) and associated tumors (red symbols). Data is derived from Oncomine as described in the methods. PMM = Pleural Malignant Mesothelioma. Embry. Carcinoma = Embryonal Carcinoma. Bladder cancer = Superficial Bladder Carcinoma. Gastric cancer = Stomach Adenocarcinoma. Gas. Mix. Adenocarc. = Gastric Mixed Adenocarcinoma. Gas. Intest. Type. Adenocarc = Gastric Intestinal Type Adenocarcinoma. Diffuse Gastric Adenocarc. = Diffuse Gastric Adenocrcinoma. BCC = Basal Cell Carcinoma. SCC = Squamous Cell Carcinoma. Melan.=Melanoma. (**B**) Scatter plots of ADCY10 mRNA expression (represented as Log2 median centered ratio) in normal controls (black symbols) and associated tumors (red symbols). Data is derived from Oncomine as described in the methods. RCC = Renal Cell Carcinoma. SCC = Squamous Cell Carcinoma. (**C**) Scatter plots of ADCY10 mRNA expression (represented as Log2 median centered ratio) in normal controls (black symbols) and associated tumors (red symbols). Data is derived from Oncomine as described in the methods. NPCBL = Naive Pregerminal Center B-Lymphocytes. MCL = Mantle Cell Lymphoma. B Lymph.=B Lymphocytes. HCL = Hairy Cell Leukemia. DBCL = Diffuse B Cell Lymphoma. LCL = Large Cell Lymphoma. Act. = Activated. GC = Germinal Center. Error bars represent mean ± s.e.m. Student's *t*-test. *P* values as indicated or *$P<0.05$, **$P<0.01$, ***$P<0.001$, ****$P<0.0001$.

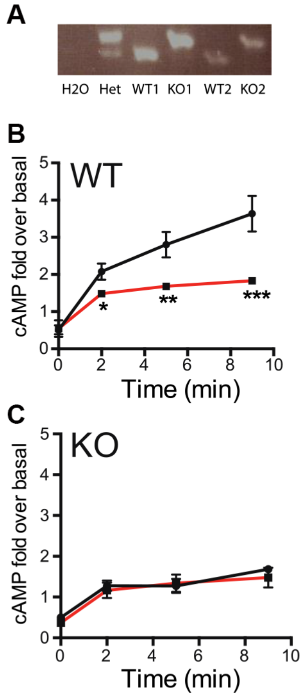

Supplementary Figure S2: Functional characterization of sAC KO cells. (**A**) To confirm the genotype of the primary fibroblasts we performed PCR as previously described (Hess et al., 2014). (**B**–**C**), Wild type cells (B) led to more cAMP accumulation over time as compared to sAC KO cells (C) and only wild type cells were sensitive to the sAC-specific inhibitor KH7 (50 µM, red lines) as compared to DMSO control (black lines). cAMP accumulation was performed as previously described(see supplemental reference 1).

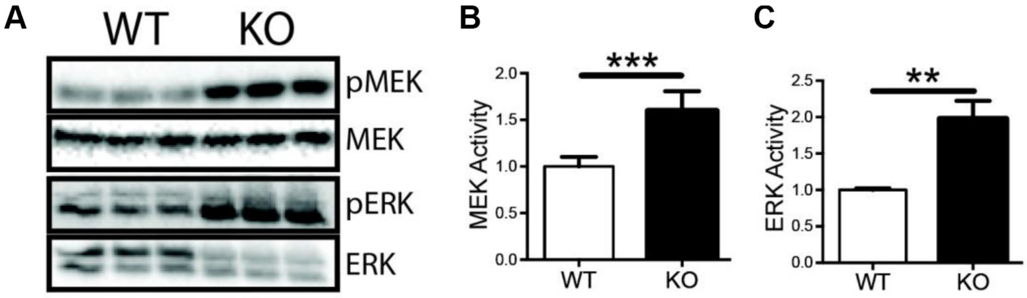

Supplementary Figure S3: Loss of sAC activity leads to MAPK pathway activation in a cAMP-dependent manner. MAPK activity in SV40 MEF cells grown in the presence of serum. (**A**) Western blot and (**B**–**C**) quantitation (normalized to WT) of (B) pMEK/MEK (MEK Activity, 43 kDa) and (C) pERK/ERK (ERK Activity, 44/42 kDa) in SV40 WT (white bars) and SV40 sAC KO (black bars) For A-C, $N = 3$. (B–C) Student's *t*-test, error ± SEM. $^{**}P < 0.01$, $^{***}P < 0.001$.

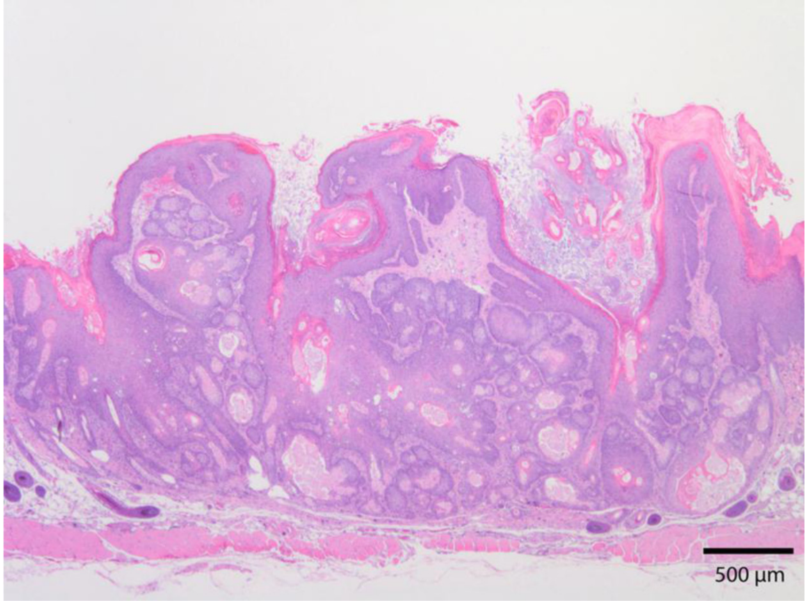

Supplementary Figure S4: Squamous cell carcinoma in wild type mice. Hematoxylin and eosin stained section of a representative example of a squamous cell carcinoma (SCC) from a wild type mouse following DMBA/TPA treatment. Image demonstrates full thickness epidermal atypia.

Supplementary Table S1: Histologic evaluation of DMBA/TPA-induced tumors in wild type and sAC KO mice

Mouse ID	genotype	Sex	Weight (g)	Mass #	Histologic diagnosis of skin mass
973	WT	F	22.9	1	SCC
				2	no histo performed
977	WT	F	22	1	no histo performed
				2	papilloma
980	WT	F	22	1	no histo performed
				2	no histo performed
				3	papilloma
				4	papilloma
				5	no histo performed
999	WT	F	21.1	1	papilloma
2286	KO	M	25.96	1	papilloma
				2	papilloma
				3	papilloma
				4	papilloma
				5	papilloma
				6	papilloma
				7	papilloma
				8	papilloma
				9	papilloma
				10	papilloma
				11	papilloma
				12	papilloma
				13	papilloma

				14	papilloma
				15	papilloma
				16	papilloma
				17	papilloma
1614	KO	F	22.37	1	SCC
				2	SCC
				3	papilloma
1301	KO	F	22.45	1	papilloma
				2	papilloma
				3	papilloma
				4	no histo performed
2280	KO	F	22.84	1	SCC
2289	KO	F	22.19	1	SCC
				2	papilloma
958	WT	F	20.62	1	papilloma
				2	papilloma
970	WT	F	21.33	1	papilloma
971	WT	F	21.72	1	Benign Sebaceous tumor (seboacanthoma)
				2	Benign Sebaceous tumor
				3	papilloma
				4	SCC
948	WT	M	28.03	1	papilloma
				2	papilloma
2277	KO	M	24.68	1	papilloma
				2	SCC
				3	papilloma
				4	papilloma
				5	papilloma
				6	papilloma
				7	papilloma
				8	papilloma
2285	KO	M	26.05	1	papilloma
				2	SCC
				3	papilloma
				4	papilloma
				5	SCC
				6	SCC
2283	KO	M	26.59	1	papilloma
				2	SCC
				3	papilloma
				4	papilloma
				5	papilloma
2310	KO	M	25.49	1	papilloma
				2	papilloma
				3	papilloma
2278	KO	M	24.64	1	papilloma
				2	Mastocytoma

				3	papilloma
927	WT	M	28.24	1	papilloma
2311	KO	F	24.48	1	papilloma
				2	papilloma
				3	papilloma
942	WT	M	29.02	0	
947	WT	M	27.68	0	
953	WT	M	25.55	0	
2298	KO	F	22.09	1	SCC
2297	KO	F	23.31	1	SCC
2317	KO	F	23.68	1	Benign Sebaceous tumor (seboacanthoma)
914	WT	M	29.8	0	
915	WT	M	29.36	0	
934	WT	M	29.83	0	
2294	KO	M	24.89	0	

REFERENCES

1. J. L. Bitterman, L. Ramos-Espiritu, A. Diaz, L. R. Levin, J. Buck, Pharmacological distinction between soluble and transmembrane adenylyl cyclases. The Journal of pharmacology and experimental therapeutics. 2013; 347:589–598.
